# Innovation in Healthcare Education: Using “Shark Tank” Approaches to Educate Teams of Interprofessional Students in Health Equity

**DOI:** 10.1007/s40670-024-02042-8

**Published:** 2024-05-02

**Authors:** Omolola E. Adepoju, Mary E. Tipton, Lauren R. Gilbert

**Affiliations:** 1 Department of Health Systems and Population Health Sciences, Tilman J Fertitta Family College of Medicine, University of Houston, Houston, TX USA; 2 Humana Integrated Health Systems Sciences Institute, University of Houston, Houston, TX USA; 3 Division of Kinesiology and Health, University of Wyoming, Laramie, WY USA

**Keywords:** Interprofessional; Medical education, Population health, Health equity

## Abstract

Inspired by the television show *Shark Tank*, we developed a summer research program that brought together students from the Colleges of Medicine, Pharmacy, Business, and Communications, to collaborate, find creative solutions, and spark innovations in population health. Under the guidance of faculty, student teams conducted literature reviews and data-thon exercises to examine trends to identify health equity gaps. Students then worked collaboratively to develop and pitch innovative solutions in front of a panel of expert “sharks” for feedback and to gain financial support to advance their ideas.

## Background

Innovation, the adoption and spread of new ideas and services to improve quality of services and life [1, 2], is a well-known challenge in the healthcare field [3]. With the renewed focus on patient-centeredness and value-based care, the need for workforce innovations in integrated care is gaining traction. Despite these demands and purported benefits, others have described the major barrier to innovation as not the scarcity of innovation, but the non-rapid diffusion [4] and dissemination of innovative concepts [5]. This translation lag suggests the need to expose future healthcare teams, at an early stage in training, to learning models that offer interdisciplinary approaches to patient care.

Prior work suggests that medical students perceive innovations as grand-scale, unapproachable feats. Students in a study by Mylopoulos et al. expressed the belief that innovation was beyond their scope of responsibilities, suggesting that the practice was exclusive to experts in the field [6]. With most students primarily concerned about acquiring and applying extensive amounts of new knowledge [7], students rejected their own problem-solving abilities, citing their ideas to be inferior to their more expert colleagues [6]. The students viewed innovation as something they would eventually engage in with acquired knowledge and experience [6]. Nevertheless, experts suggest the need to expose learners to innovation early in their academic training, as a way of acclimatizing them to the “real-world” post-graduation.

To address this, many medical schools have integrated project-based learning into their curriculum [8], with a review by Arais et al. noting lectures and capstone projects as the most common metrics among innovation and entrepreneurship trainings in medical programs [9]. However, prior evaluation suggests that these approaches yield mixed results, with Levitt et al. reporting no significant improvement on student knowledge of quality improvement (QI) concepts following a year-long integrated third year clerkship [10]. This sub-optimal outcome highlights the need to reflect the challenges and core values of innovation, how to use evidence to drive solutions while working with interprofessional teams, and experienced mentors [11].

Inspired by the television show *Shark Tank*, we developed a summer research program that brought together interprofessional students to find creative solutions and spark innovations in current population health topics [12, 13]. This work builds on the social constructivist learning theory, which defines learning as an interdependent act that builds on the social interactions learners have with one another [14]. Under the guidance of faculty, student teams conducted literature reviews and data-thon exercises to quantify the issue and presented solutions [15] in front of a panel of expert “sharks” for feedback and to gain financial support to advance their ideas [16]. Recognizing the importance of a collaborative environment between students, the objective of this format was to create a safe and energizing forum to encourage innovation [17] and give students adequate resources and advising needed to pilot these innovative ideas, with steady, consistent mentorship [18].

## Activity

**Fig. 1:**
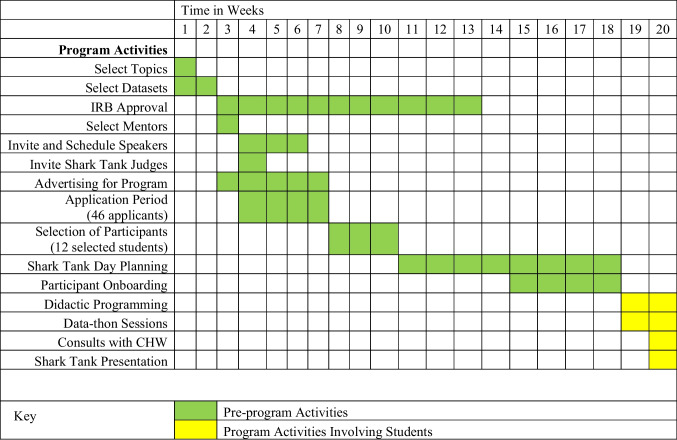
Planning and program execution timeline

The program was planned and executed over 20 weeks (Fig. 1). Program leaders pre-selected three topics, based on pressing public health issues: (1) disrupting current opioid use disorder (OUD) treatment patterns; (2) leveraging ChatGPT to promote patient engagement among underserved populations; and (3) strategies to slow diabetes progression. Students were asked to complete an application for the program, indicating demographic characteristics and educational background. Applicants were asked to select one topic, providing a 300-word narrative justifying their selection. A final prompt asked applicants to indicate their experience and views on interprofessional teams. Flyers to advertise the program were distributed via social media and emails to student advisors across colleges and student-led organizations.

**Fig. 2:**
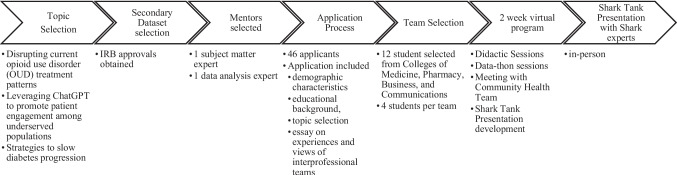
Shark Tank team composition

Completed applications were blinded, scored, and ranked by faculty, using a rubric. Of the 46 students who applied, 12 were selected. The small number was due to limited funding to test proof of concept. The final teams consisted of three groups of four students (see Fig. 2 for team composition). Selected students represented the Colleges of Medicine, Pharmacy, Business, and Communications, ensuring diverse perspectives in the creation of realistic and well-rounded solutions. Due to scheduling constraints, all medical students were between their first and second years of training. Graduate students from other colleges were a mix of master’s and doctoral students.

For each topic, secondary data sources were pre-identified for teams to use, and students were added to the institutional review board (IRB) protocol for their respective studies. All IRB submissions were completed by program leaders, and approvals obtained before the start of the program. After IRB approval, program leaders identified faculty mentors with expertise in data analytics, community-based participatory research, epidemiology, health services research, and public health to serve as mentors to the student teams. Each topic had two mentors: one had subject-matter expertise while the other had data analysis expertise. The entire administrative planning process lasted 20 weeks and was completed prior to program initiation. Each student received a $1000 stipend for their participation in the program and competition.

The program occurred primarily virtually over 2 weeks in the summer. Each day commenced with an hour-long didactic session led by industry experts. Topics included “Technology and Health Disparities,” “Caring for the Underserved,” and more. In the second half of the morning, designated as the “Data-Thon” sessions, students engaged with their mentors to delve into their datasets. During the data-thon sessions, students engaged in applied data analyses, covering topics such as “PICO Research Questions,” “Univariate Analysis,” and “Adjusted Models.” These active data sessions exposed students to Excel, R, ArcGIS, and Stata statistical software. Students also met with a community health worker team to ensure their solutions were culturally appropriate to the communities they targeted.

At the end of the virtual 2 weeks, teams pitched their evidence-based solution to an in-person expert panel using a “Shark Tank” format. Shark selection was strongly based on the three topics. Faculty identified five non-university affiliated health systems leaders with respective expertise on substance use (Meninger Clinic), health technology (Microsoft ChatGPT and Care Message), and diabetes care (Humana, Veterans Administration). Invitations were sent 3 months before the start of the program. Sharks were not compensated monetarily, and they did not provide any financial incentives for the solutions proposed by the students.

After the presentation, each team had a public dialogue with the Shark panel where they fielded probing questions into the feasibility, viability, and impact of their proposed solutions. To remove bias from the judging, student presentations were judged using a rubric weighing the quality of the presentation, scientific content, innovation, and feasibility, which were averaged together to create a ranked score. In addition to formal recognition, the winning team received travel funds to present their work at a conference.

## Results and Discussion

In addition to having interprofessional diversity, student participants were also racially and gender diverse. (See Table 1.) In total, 74% reported having no prior experience in interprofessional teams before enrolling in the program. Of the students, 100% agreed to the sentence “I learned information in the summer program that will be useful to me in my future career” and 100% indicated that they would recommend the program to peers. Qualitative feedback provided by students includes:

I really appreciated how the program fosters interdisciplinary research to better health outcomes for the local community. This opportunity allowed me to learn so much and apply my unique finance and tech background in health equity.

I enjoyed every part of this program: from our morning sessions to data-thon, to our final presentations. If given the opportunity, I would do it all over again

**Table 1:** Characteristics of student participants in the “Shark Tank” program (*n* = 12)

**Variables**	**Total** ***n* = 12**	
	***n***	**(%)**
**Age (mean, SD)**		
	26.7	(3.4)
**Gender**		
Female	8	(66.0)
Male	4	(44.0)
**Race/ethnicity**		
Non-Hispanic Black	3	(25.0)
Hispanic	3	(25.0)
Asian	5	(40.0)
Not specified	1	(10.0)
**College affiliation**		
Medicine	7	(58.0)
Business	3	(25.0)
Pharmacy	1	(8.0)
Communications	1	(8.0)
**Interprofessional experience**		
No	9	(74.0)
Yes	3	(26.0)

All three teams continued scholarly activity beyond the conclusion of the program, with each submitting their work for presentation at national and regional conferences. The teams are currently looking for opportunities to pilot test their solutions with community partners. The current teams and subsequent participants will continue to be tracked to evaluate the impact of this program. This experience was largely virtual, except for the last 2 days of the program, suggesting geography need not be a barrier to interprofessional healthcare teams. Furthermore, the success of the program suggests that with appropriate guidance, both non-physicians and medical students can create reasonable, accessible solutions.

As with our program, “Shark Tank”–inspired programs have seen success in a multitude of settings. For example, since 2014 Johns Hopkins has hosted a small grant program modeled after the show, which has kickstarted projects to improve training for students entering surgical fields and ambulatory curriculum [12]. On a larger scale, in 2015 the VA launched a national competition modeled after the show to encourage employee innovations [19, 20]; it has led to impactful programs like improving population health via telemonitoring [21], reducing costs for caregivers [22], and improving staff satisfaction [19]. These results show the potential that “Shark Tank”–inspired programs can have on both students and industry.

We conclude that short, intensive, and interprofessional research experiences can be used to teach data analysis skills with subsequent development of culturally appropriate, evidence-based solutions to advance healthcare. Importantly, the innovative approach used for the program allows students to practice in an interprofessional setting, and hone their critical thinking and data analysis [23, 24] skills—skills that are fundamental to operating practices and optimizing patient outcomes [6].

## Data Availability

Data sharing not applicable to this article as no datasets were generated or analyzed during the current study.
